# Sputum and serum hydrogen sulfide (H_2_S) as novel biomarker of asthma

**DOI:** 10.1186/2045-7022-3-S1-P3

**Published:** 2013-05-03

**Authors:** Junpei Saito, Pankaj Bhavsar, Qingling Zhang, Christopher Hui, Andrew Menzies-Gow, Kian Fan Chung

**Affiliations:** 1 Experimental Studies, National Heart and Lung Institute, Imperial College London & Biomedical Research Unit, Royal Brompton Hospital, London, UK

## Background

Hydrogen sulfide (H_2_S) is a gas produced by respiratory cells including smooth muscle cells and may play a role as a gasotransmitter. We determined whether H_2_S levels in serum or sputum supernatants could represent a biomaker of asthma.

## Methods

We measured H_2_S in induced sputum and serum samples of patients with severe and non-severe asthma and of healthy subjects. H_2_S concentrations were measured using a sulfide-sensitive electrode.

## Results

H_2_S levels in induced sputum from severe and non-severe asthmatic patients were significantly higher than those from healthy subjects but there was no difference between the severe and non-severe group. Serum H_2_S levels were 10 times higher than in sputum and these were also higher in severe and non-severe asthmatic subjects compared to healthy subjects. There was a positive correlation between sputum and blood H_2_S levels (r=0.42, p<0.05). Sputum H_2_S levels were negatively correlated with FEV_1_ %predicted (r=-0.42, p=0.003), and with reversibility to salbutamol (r= -0.54, p<0.01). There was a correlation between sputum H_2_S and sputum neutrophils and macrophages, and a negative correlation between sputum H_2_S and Fe_NO_ levels.

## Conclusions

Endogenous H_2_S, measured in induced sputum, may be a marker of neutrophilic inflammation and bronchial narrowing.

